# Contrary to expectation: The surface urban heat island intensity is increasing in population shrinking region while decreasing in population growing region-A comparative analysis from China

**DOI:** 10.1371/journal.pone.0300635

**Published:** 2024-03-18

**Authors:** Luofu Liu, Wei Zhang

**Affiliations:** 1 School of Earth and Environmental science, The University of Queensland, Queensland, Australia; 2 School of Geographical Sciences, Southwest University, Chongqing, China; Chongqing University, CHINA

## Abstract

Exploring the complex relationship between population change and surface urban heat island (SUHI) effect has important practical significance for the ecological transformation development of shrinking cities in the context of the prevalence of urban shrinkage and the global climate change. This paper compares the population change and SUHI effect between population shrinking region (Northeast Region, NR) and population growing region (Yangtze River Delta, YRD) in China, and explores their differences in driving mechanisms, using GIS spatial analysis and Geodetector model. Our results indicated that there are significant differences in population changes and SUHI intensity between these two regions. About 72.22% of the cities in the NR were shrinking, while their SUHI intensities increased by an average of 1.69°C. On the contrary, the urban population in the YRD shows a linear growth trend, while their SUHI intensities decreased by 0.11°C on average. The results of bivariate Moran’s I index also indicated that the spatial correlation between the urban population changes and the SUHI intensity changes are not significant in the above regions. Furthermore, there are significant differences in the primary drivers of SUHI variations between these two regions. In the NR, underlying surface changes, including the changes of green coverage and built-up areas, are the most important driving factors. However, atmospheric environment changes, such as carbon dioxide emission and sulfur dioxide emission, are the key drivers in the YRD. Northam’s theory of three-stage urbanization and environmental Kuznets curve hypothesis are powerful to explain these differences.

## 1. Introduction

Both growth and shrinkage are essential historical stages in the process of urbanization [1, 2]. However, the growth-oriented mode occupied the mainstream of urban development theory since the Industrial Revolution in the middle of the 18th century [3]. Urban shrinkage has usually been ignored in this context because the antithesis of growth, such as population loss, economic slowdown and land vacancy, are often negatively labeled as “failure”, “recession” and “ignominy”. In recent years, urban shrinkage becomes a hot topic due to the prevalence of shrinking cities. Nearly a quarter of the world’s cities are experiencing long-term population decline because of the combined effect of globalization, deindustrialization, suburbanization and population aging [4]. Cities in America’s rust belt, such as Detroit and Pittsburgh, have lost even more than half of its population between 1960 and 2010 [5, 6]. Growing attentions have been paid on urban shrinkage because it is recognized as a global phenomenon and affects the fate of many cities decisively [7, 8].

Urban heat island (UHI) effect is the phenomenon that urban areas are warmer than their rural hinterlands [9]. In recent years, growing greenhouse gas emissions and accelerating urbanization process have caused the rising of global temperature [10], and then triggered the increasing of extreme heat events and the aggravation of UHI effect. Against this background, the health threats of extreme heat events, UHI effect and their synergy on urban residents are growing [11]. For example, the 2003 heat wave in France caused about 15,000 excess deaths [12]. Heat waves have been listed as the most dangerous natural disasters in many places like the United States, Europe and Australia [13]. The mitigation of UHI effect has become a hot topic globally.

Urban shrinkage was usually regarded as a threat or a challenge in earlier studies [14]. Recently, growing scholars begin to explore the opportunities brought by urban shrinkage, especially for the potential ecological opportunities. Haase et al. [15] believed urban shrinkage offers great potential for rebuilding urban green space and they developed a matrix approach to link population shrinkage and ecosystem services. Zeng et al. [16] found that urban shrinkage helps the improvement of CO_2_ emission efficiency. Unfortunately, limited literatures discussed the relationship between population shrinkage and SUHI effect, and most of them are case studies that focused on individual cities. For example, Emmanuel and Krueger [17] discussed the variation of the UHI intensities in the urban shrinkage and growth processes in Glasgow, UK. Jang [18] explored the impacts of demolition programs for abandoned houses on thermal comfort in Daegu, South Korea. Cai et al. [19] analyzed the impact of short-term population loss on urban thermal environment in Wuhan, China. These case studies provide important references for subsequent research. Nevertheless, it is difficult to obtain universal conclusions from a small number of case studies considering the diversity of shrinking cities. Peng et al. [20] explored the evolution characteristics of SUHI effect in 180 shrinking cities in China. But they did not comprehensively compare the SUHI effects of shrinking and growing cities, nor did they quantify its driving mechanisms. In addition, urban shrinkage and SUHI effect are closely related to the regional background of each city. However, previous studies have not analyzed the relationship between population shrinkage and SUHI effect from the regional perspective. In summary, exploring the relationship between urban shrinkage and SUHI effect at the regional scale is helpful to fill these research gaps. Its significances are as follows: (1) it helps to reveal the influence of population shrinkage on the urban eco-environment, and thus provides some empirical evidences for the ecological transformation development of shrinking cities [15, 16]. (2) It is helpful to unravel the complex relationship between population change and SUHI effect [19, 20], so as to provide useful assistance for the mitigation of SUHI effect during the population shrinkage process. (3) Most previous studies on urban shrinkage and UHI effect were focused on individual cities [5, 17, 19]. The regional perspective presented in this study is hopeful to make a good contribution to the literatures.

In this paper, a population shrinking region (Northeast Region) and a growing region (Yangtze River Delta) in China were selected to explore the relationships between urban population shrinkage and SUHI intensity. This paper seeks to address the following questions: (1) are there any differences in SUHI effects between a typical shrinking region and a growing region? (2)Which factors are the key drivers of their respective SUHI effects? (3) How to mitigate the SUHI effect in the context of regional population change?

## 2. Material and methods

### 2.1. Study area

China has experienced an unprecedented process of rapid urbanization since the 1980s. Its urban population increased from 191 million in 1980 to 914 million in 2021; and its urban built-up area has also increased from 7,438 km^2^ in 1980 to 62,421 km^2^ in 2021 [21]. However, China’s rapid urbanization has also given rise to a series of eco-environmental problems, including UHI effect [22]. The UHI effect in China is continuously increasing due to the combined effect of multiple factors like climate change, built-up area expansion, population agglomeration and air pollution, which poses a serious threat to the health of urban residents. The results of previous studies indicated that the heat-related deaths in China increased fourfold between 1990 and 2019 [23].

Shrinking cities have emerged in China in the past few years for multiple reasons like population aging, fewer children and economic fluctuation [24]. The Northeast region (NR) is the most typical shrinking region because it has the most shrinking cities in China [25]. From 2010 to 2020, the total population of the NR decreased by 11.3 million [21]. On the contrary, the Yangtze River Delta (YRD) is a population growing region. From 2010 to 2020, the total population of the YRD increased by 19.62 million [21]. YRD is one of the most developed regions in China. It contributes nearly a quarter of China’s economic output with less than 4% of China’s territory. To sum up, NR and YRD are the two most representative cases in China from the perspective of regional population change. It is helpful to clarify the complex relationship between regional population change and SUHI effect by the comparative analysis of these two typical regions.

### 2.2. Data sources and pre-processing

(1) MODIS Land Surface Temperature (LST) data
  MOD11A2 LST product with a spatial resolution of 1km was used to analyze the SUHI effect in China. The original remote sensing image of this product was produced by the Moderate-resolution Imaging Spectroradiometer (MODIS), and this product can be downloaded on the official website (https://ladsweb.modaps.eosdis.nasa.gov/) of the National Aeronautics and Space Administration (NASA). MODIS Reprojection Tool (MRT) provided by the Land Processes Distributed Active Archive Center (LP DAAC) (https://lpdaac.usgs.gov/lpdaac/tools/) was utilized for the preprocessing of MODIS products like trans-projection, splicing and clipping. The value of 0 in the quality assurance layer was utilized to filter the MOD11A2 pixels for accuracy guarantee. The time resolution of MOD11A2 LST product is 8 days, so the maximum value compositing method [13] was used to generate annual LST data for 2010–2020.
(2) Meteorological data
  Annual spatial interpolation data of multiple meteorological elements, including sunshine hours, average wind speed, relative humidity and evapotranspiration, were obtained from the Resource and environment science and data center, Chinese Academy of Sciences (https://www.resdc.cn/). This data was produced from the daily observation data of corresponding meteorological elements at more than 2400 meteorological stations in China, using Anuspl software for spatial interpolation. The spatial resolution of this data is 1 km.
(3) Digital Elevation Model (DEM) data
  The Advanced Spaceborne Thermal Emission and Reflection Radiometer (ASTER) Global Digital Elevation Model (GDEM) V002 dataset used in this paper was obtained from the official website of NASA (https://search.earthdata.nasa.gov/search/). Its spatial resolution is 30 m. ArcGIS 10.5 software was utilized to obtain the average elevation of each city.
(4) Normalized difference vegetation index (NDVI) data
  Monthly NDVI data (2010–2020) with a spatial resolution of 1km was obtained from the National Earth System Science Data Center, National Science & Technology Infrastructure of China (http://www.geodata.cn). Maximum value compositing method was used to generate annual NDVI data for 2010–2020.
(5) Land cover type data
  Annual land cover type data for 2010–2020 with a spatial resolution of 30 m was obtained from Wuhan University. Its overall accuracy reached 79.31%. The land cover types of this data include cropland, forest, shrub, grassland, water, snow and ice, barren, impervious, and wetland. More details about this data can be found in Yang and Huang [26].
(6) Fine particulate matter data
  Annual particular matter with a diameter smaller than 2.5 microns (PM_2.5_) data for 2010–2020 was obtained from the Atmospheric Composition Analysis Group, Washington University in St. Louis (https://sites.wustl.edu/acag/datasets/surface-pm2-5/). Its version is V5.GL.03, and its spatial resolution is 0.01° × 0.01°. More details about this data can be found in Aaron et al. [27].
(7) Carbon dioxide emission data
  Carbon dioxide (CO_2_) emission data for 2010–2020 was derived from the Open-Data Inventory for Anthropogenic Carbon dioxide (ODIAC), which is produced by the center for global environment research, national institute for environmental studies (https://db.cger.nies.go.jp/dataset/ODIAC/). The spatial resolution of this data is 1 km, and its version is ODIAC2022. More details about this data can be found in Oda et al. [28].
(8) Statistical data
  Statistical data at the city scale was used to construct multiple variables in this paper, including urban population, urban built-up area, GDP, volume of sulphur dioxide emission, number of industrial enterprises above the designated size, etc. These statistics were obtained from the China City Statistical Yearbook and the China Urban Construction Statistical Yearbook.

### 2.3 Methodology

#### 2.3.1. Calculation of SUHI intensity

According to the concept of UHI effect, SUHI intensity was defined as the temperature difference between urban area and its surrounding rural area [29, 30]. Two land cover types, urban impervious surface and rural cropland, were selected as typical urban and rural area respectively in this research [31]. The reasons for selecting arable land as the rural reference sites are as follows: (1) Arable land is a typical landscape in rural areas. (2) Compared with arable land, forest and wetland are less affected by humans. However, the surface temperatures of forest and wetland were significantly lower than others, so usually they were eliminated from normal rural areas for avoiding the overestimate of the SUHI effect [29, 31]. (3) The study area of this paper is located in the northeast and eastern regions of China, while the grassland and bare land are concentrated in the western region of China. Therefore, they are unsuitable for this research.

**Fig 1:**
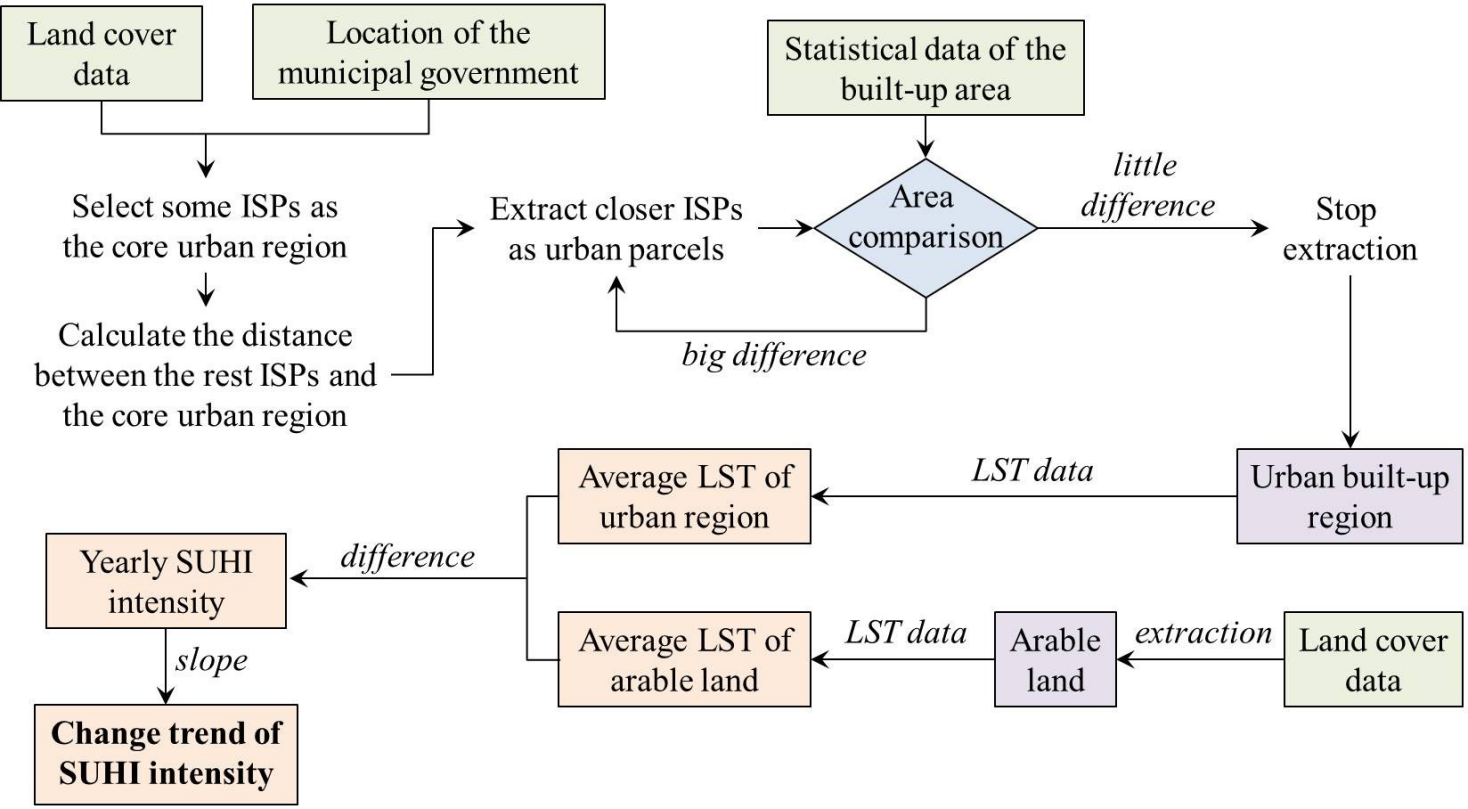
Technical flow chart of the calculation and analysis of SUHI intensity. Abbreviation. ISP: impervious surface parcel; LST: land surface temperature; SUHI: surface urban heat island.

The SUHI intensity of each city was calculated and analyzed by the following steps (Fig 1). (1) Referring to the location of the municipal government, several large-size and contiguous impervious surface parcels (ISPs) were selected as the core urban region of each city. (2) The Near tool provided by ArcGIS 10.5 software was used to calculate the distance between the rest ISPs and the core urban region. (3) Divide the rest ISPs into urban/rural parcels according to the calculated distance and the built-up area data of each city provided by China City Statistical Yearbook. The ISPs closer to the core urban region will get a higher priority to be extracted as urban parcels. If the total area of the extracted urban ISPs is close to the statistical data of each city, this extracting process will complete. (4) Counting the average LST value of each city’s impervious surface and cropland by ArcGIS 10.5 software, using MOD11A2 LST data and the spatial data of urban built-up region and surrounding arable land. (5) Calculating the yearly SUHI intensity of each city with the following formula.

$$SUHI_{ij}=LSTU_{ij}-LSTR_{ij}$$ (1)

where *SUHI*_*ij*_ represents the SUHI intensity of city *i* in year *j*; and a bigger value of *SUHI*_*ij*_ means stronger SUHI effect. *LSTU*_*ij*_ and *LSTR*_*ij*_ respectively represent the average LST of urban area and rural area of city *i* in year *j*.

(6) The mathematical concept of “slope” was introduced to represent the dynamic trend of SUHI intensity in each city. The fitting formula is as follows:

$$Y_{i}=k_{i}X+b$$ (2)

where *Y*_*i*_ is the SUHI intensity of city *i*; *k*_*i*_ represents the slope of the corresponding fitted trend line of city *i*, *X* represents the year, and *b* is the intercept term. When *k*_*i*_< 0, it means that the change trend of SUHI intensity for city *i* is decreasing during the study period; and vice versa. Excel software was utilized to linearly fit change trends of the SUHI intensity of each city during 2010–2020.

In addition, *k*_*i*_ in formula (2) was used to represent the dynamic trend of urban population too. Cities with a value of *k*_*i*_ less than 0 were classified as shrinking cities.

#### 2.3.2. Statistics test method

Parametric test and nonparametric test tools were utilized to test the significance of regional difference in population change and SUHI effect between NR and YRD. Specifically, the Independent-samples T test was selected for parameter testing; and Mann-Whitney U test, Kolmogorov-Smirnov Z test and Wald-Wolfowitz Runs test were selected for nonparametric testing. SPSS 25.0 software was used to complete these statistics tests.

#### 2.3.3. Spatial correlation analysis

Statistics test methods cannot reflect the spatial relationships among the samples. Therefore, global bivariate Moran’s I index was utilized to explore the spatial correlation between urban population and SUHI effect. It was calculated by the following formula [8]:

$$MI_{ab}=\frac{n\sum_{i}^{n}\sum_{j\neq 1}^{n}W_{ij}Z_{i}^{a}Z_{j}^{b}}{\left(n-1\right)\sum_{i}^{n}\sum_{j\neq 1}^{n}W_{ij}}$$ (3)

where *MI*_*ab*_ represents the global bivariate Moran’s I index of urban population (variable *a*) and SUHI intensity (variable *b*), and its value range is [-1, 1]. *MI*_*ab*_ >0 indicates these two variables (*a* and *b*) are spatially positively correlated, and *MI*_*ab*_ <0 means negatively correlated. *W*_*ij*_ represents the distance weight matrix; *n* is the number of spatial units; $Z_{i}^{a}$ and $Z_{j}^{b}$ represent the normalized values of the proportion of variables *a* and *b* in spatial units *i* and *j* respectively. GeoDa 1.14.0 software was used to complete the calculation.

#### 2.3.4. Geodetector model

Geodetector is a statistical model to detect spatial stratified heterogeneity and reveal the driving factors behind it [32]. In this research, Geodetector model was utilized to identify the key driving factors of SUHI effect and to detect the interaction of drivers. In this model, *q* statistic is a crucial output to judge the explanatory power of independent variables (*X*_*i*_) to the dependent variable (*Y*). The formula of *q* statistic is as follows.

$$q=1-\left(\sum_{h=1}^{\text{L}}N_{h}\sigma_{h}^{2}\right)/{\text{N}\sigma }^{2}$$ (4)

where *h* is the type quantity of the explanatory variables; and K-means clustering algorithm was utilized for the discretization of continuous variables. *N* is the total number of samples, *σ* presents the variance of samples. The value range of *q* statistic is [0, 1]; and the bigger the *q* statistic of a variable, the stronger its explanatory power. More details about Geodetector model can be found in Wang et al. [32].

#### 2.3.5 Driving mechanism analysis

**Fig 2:**
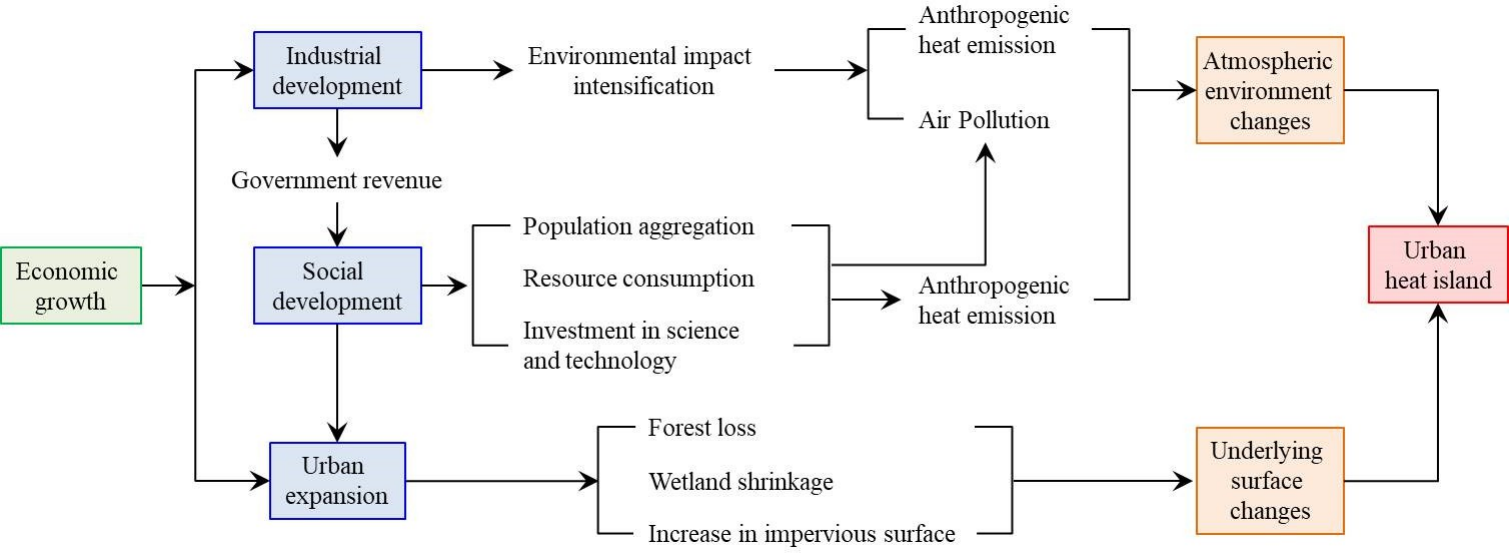
Driving mechanism of socio-economic factors on SUHI effect.

The forming mechanism of SUHI effect is complicated. Numerous social, economic and environmental factors are contributed to SUHI effect. This research focuses on the following socio-economic factors considering its research objectives (Fig 2).

(1) Population change. Urban population change exerts multiple effects on SUHI intensity. First, residents will directly increase the urban heat through human metabolism and energy consumption in their daily life. These anthropogenic heat emissions are important contributors to SUHI effect. Previous studies have proved that even temporary urban population reduction can reduce SUHI intensity immediately [33, 34]. Secondly, urban residents will indirectly affect the SUHI intensity through their impact on land cover change [35, 36].
(2) Underlying surface change. Compared with rural regions, the space in the city is very limited. Consequently, drastic land cover change is an inevitable part of urbanization process. The rapid increase in impervious surface raises the temperature of urban regions because its heat capacity is small, while heat conductivity is large [37, 38]. Meanwhile, forest and wetland have important ecological functions of local microclimate regulation. Unfortunately, they are significantly shrinking during the urbanization process [39]. In short, the drastic change of underlying surface is one of the major contributors to SUHI effect.
(3) Socio-economic change. The socioeconomic development has a dual impact on SUHI effect [40]. On the negative side, industrial production increases energy consumption and heat emission remarkably [41]; the development of real estate industry accelerates the transformation of natural surface to artificial surface in urban regions. These socio-economic processes will exacerbate the SUHI effect [42]. On the positive side, the rapid economic development also contributes to the growth of fiscal revenue, which provides important financial guarantee for technological progress and environmental improvement, helping to mitigate the SUHI effect.
(4) Atmospheric environment change. SUHI effect is a local meteorological phenomenon [43], and there is a complex interaction between atmospheric environment and SUHI effect [44]. Previous studies have proved that LST is positively correlated with O_3_ concentration and negatively correlated with PM_2.5_, PM_10_, SO_2_, NO_2_ and CO concentration [45]. The improvement of atmospheric environment is helpful to reduce the urban-rural difference in incident radiation and thus mitigate the SUHI effect [46].

Finally, 21 socio-economic indicators were preliminarily selected as the driving factors of SUHI effect (Table 1). SPSS 25.0 software were utilized for pre-regression, and indicators with Variance Inflation Factor (VIF) bigger than 10 or failed the significance test were removed.

**Table 1:** Preliminarily selection of driving factors on the change of SUHI intensity.

First-level indicators		Second-level indicators			
Code	Name	Code	Name	Abbreviation	References
A	Population dynamics	A1	Urban population	POP	[34]
		A2	Population density	PD	[33]
B	Underlying surface changes	B1	Area of built district	ABD	[37, 38]
		B2	Normalized difference vegetation index	NDVI	[47]
		B3	Green coverage rate of built district	GCR	[48]
		B4	Public recreational green space per capita	GS	[49]
		B5	Road surface area per capita	RSA	[50]
		B6	Urban compactness	UC	[51]
C	Socio-economic changes	C1	Gross domestic product	GDP	[42]
		C2	Government revenue	GR	[40]
		C3	Secondary industry as percentage to GDP	SIP	[41]
		C4	Tertiary industry as percentage to GDP	TIP	[42]
		C5	Number of industrial enterprises above the designated size	IE	[52]
		C6	Investment in real estate development	RED	[53]
		C7	Total retail sales of consumer goods	CG	[54]
		C8	Expenditure for science and technology as percentage to local general public budget expenditure	EST	[55, 56]
		C9	Daily water consumption per capita	WC	[57]
D	Atmospheric environment changes	D1	Volume of sulphur dioxide emission	SO_2_	[44]
		D2	Volume of industrial particulate emission	IPE	[45]
		D3	Annual mean concentration of PM_2.5_	PM_2.5_	[36]
		D4	Volume of carbon dioxide emission	CO_2_	[58]

## 3. Results

### 3.1. Regional population change

**Fig 3:**
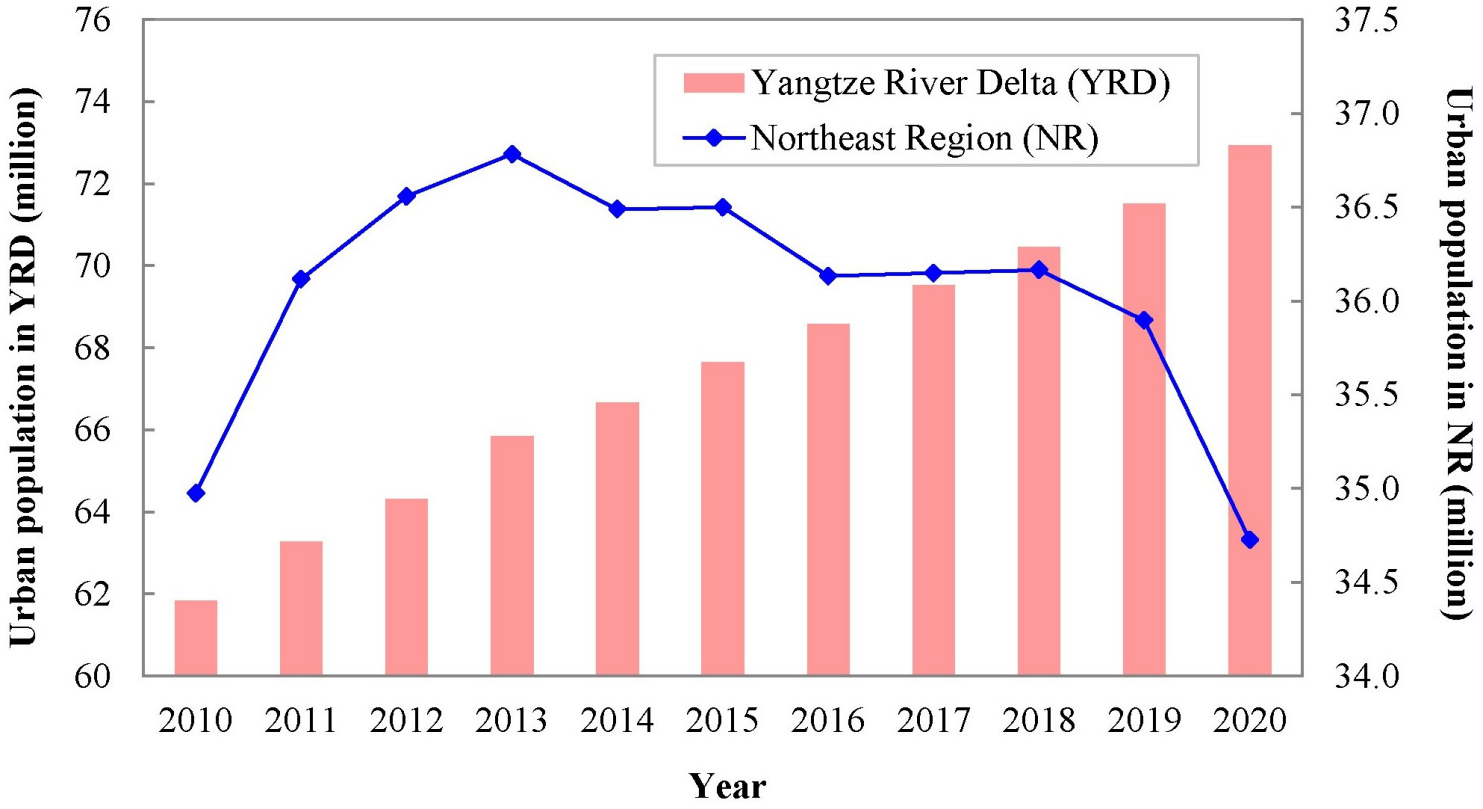
Change trends of urban population in the Northeast region and the Yangtze River Delta.

The results presented in Fig 3 indicated that the change trends of urban population in the Northeast region (NR) and the Yangtze River Delta (YRD) are quite different. The urban population of the YRD increased from 61.83 million in 2010 to 72.93 million in 2020, which shows a linear growth trend. However, the NR exhibits an inverted U-shaped trend of population change. Its urban population peaked in 2013 (36.78 million) and has continued to decline in subsequent years.

As for city scale, the number of shrinking cities in the NR is 26, accounting for 72.22% of its total number of cities. In contrast, there are only three shrinking cities in the YRD, accounting for 7.32% of its total number of cities. The result of Levene test indicated that the samples of these two regions meet the hypothesis testing of the heteroscedasticity (F = 6.233, p value = 0.015<0.05), and the corresponding results of Independent-samples T test indicated that there were significant differences in population change between these two regions (t = -4.307, Sig. (2-tailed) = 0.000 < 0.1). In addition, all the p values of three nonparametric tests, including Mann-Whitney U test, Kolmogorov-Smirnov Z test and Wald-Wolfowitz Runs test, were less than 0.01. These results manifest there are significant differences in the central location, cumulative frequency distribution curve and overall distribution of samples from these two regions.

### 3.2. SUHI intensity

The change trends of SUHI intensity in the NR and the YRD are quite different too. In the NR, the SUHI intensities at the city scale increased by 1.69°C on average during 2010–2020. Cities with rapid SUHI intensity growth were concentrated in the central part of the NR. The SUHI intensities of all the three provincial capitals, namely Harbin, Changchun and Shenyang, were increased by more than 2°C. On the contrary, the SUHI intensities in the YRD decreased by 0.11°C on average during 2010–2020. The cities with a significant increase in SUHI intensity were scattered in the peripheral areas, while the SUHI variations of provincial capital cities were not sharp.

The result of Levene test indicated that the samples of these two regions meet the hypothesis testing of the homogeneity variance (F = 1.240, p value = 0.269>0.05), and the results of following Independent-samples T test indicated that the differences of SUHI intensity changes between these two regions were not significant (t = 1.227, Sig. (2-tailed) = 0.224 >0.1). In addition, the p values of Mann-Whitney U test, Kolmogorov-Smirnov Z test and Wald-Wolfowitz Runs test were greater than 0.05, which means the differences in the central location, cumulative frequency distribution curve and overall distribution of samples from these two regions are not significant.

### 3.3 Spatial correlation

**Fig 4:**
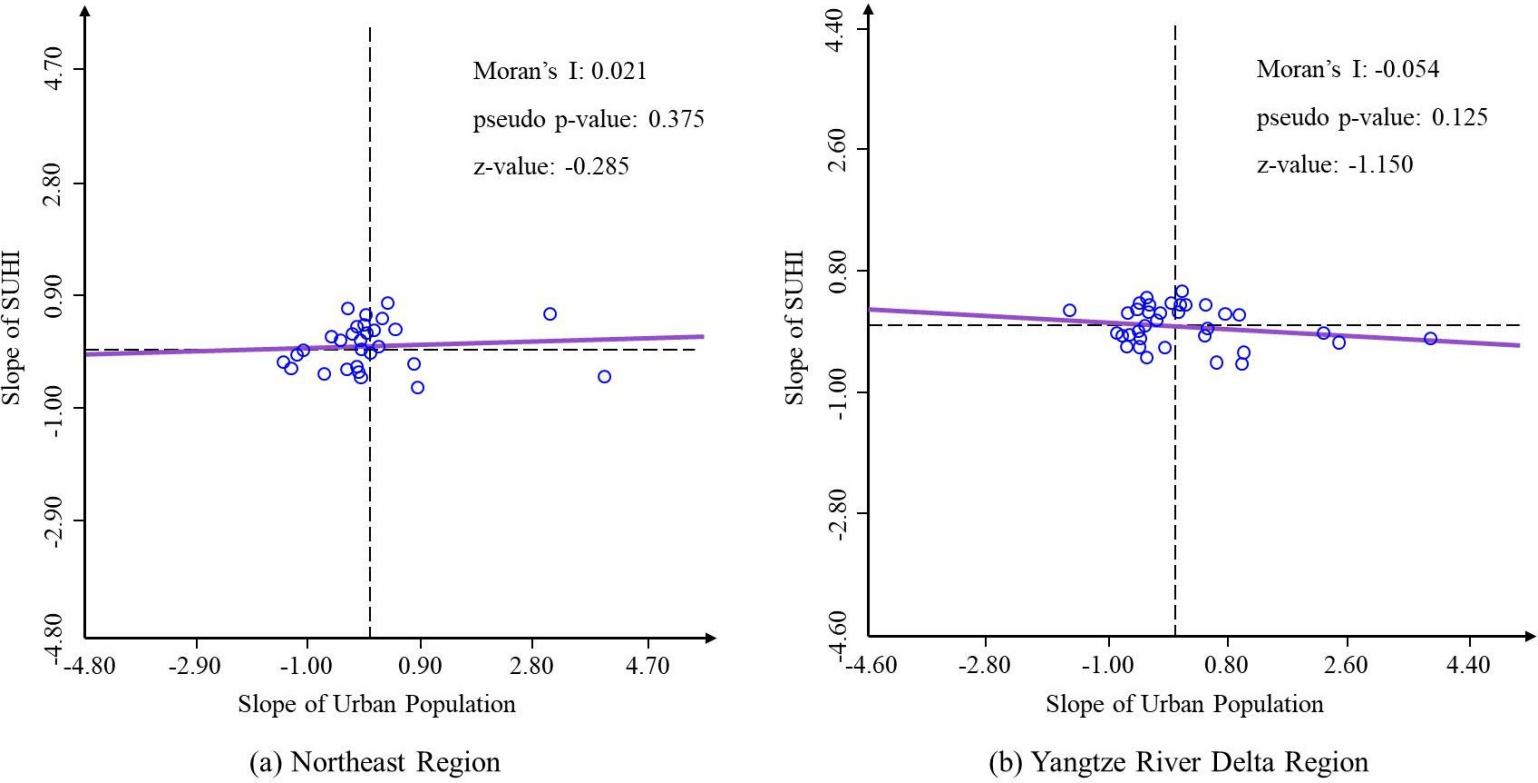
Scatter plots of global bivariate Moran’s I indexes in two regions.

As is shown in Fig 4, the global bivariate Moran’s I indexes of urban population change and SUHI intensity change of two regions are low and fail to pass the significance test (p value>0.05). These results indicate that the spatial correlation between urban population change and the variation of SUHI intensity is not significant in either population shrinking region or growing region.

### 3.4 Driving mechanism of SUHI variations

**Fig 5:**
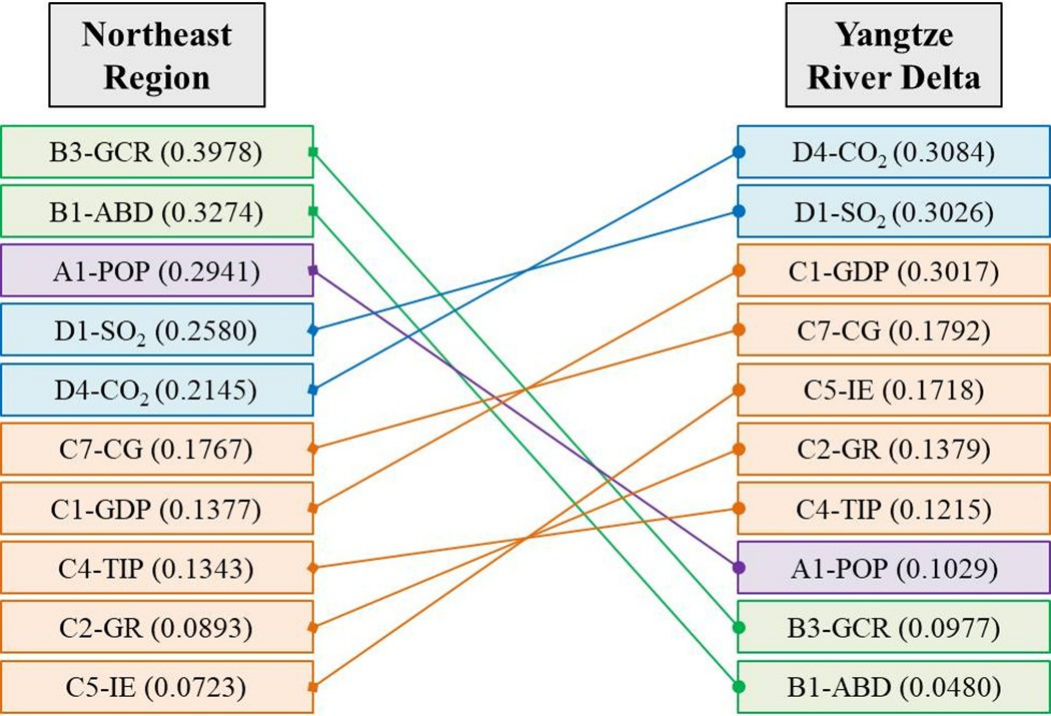
Explanatory power of each driver in two regions. Notes: (a) Values in brackets are *q* statistics; (b) The full name of each indicator can be found in Table 1.

As is shown in Fig 5, the primary drivers of SUHI variations in NR and YRD are different. In NR, the change of underlying surface is the most important driving factor, including green coverage and built-up area. In the case of Harbin, the capital of Heilongjiang province, its urban population decreased by 5.02% during 2010–2020, but its built-up area increased from 386.91 km^2^ to 473.00 km^2^, with a growth rate of 22.25%. Its per capita road surface area also increased from 7.91 m^2^ to 16.01 m^2^, with a growth rate of 102.34%. Correspondingly, its green coverage rate in built-up district decreased from 38.38% to 34.22%; and the per capita public recreational green space increased from 10.07 m^2^ to 10.19 m^2^, with a growth rate of only 1.24%. Consequently, the SUHI intensity of Harbin increased from 1.12°C to 5.69°C during 2010–2020. Shenyang, the capital of Liaoning province, is similar to Harbin. From 2010 to 2020, the built-up area of Shenyang increased by 37.62%, but its green coverage rate in built-up district decreased by 2.83%, resulting in an increase of 2.58°C in its SUHI intensity.

Atmospheric environment changes, such as carbon dioxide emission and sulfur dioxide emission, are the primary driving factor for the SUHI variation in the YRD, which is different from the NR (Fig 5). For instance, Nanjing is the capital of Jiangsu province and one of the traditional “big four stoves” in China. Its urban population and built-up area increased by 1.59 million and 249.64 km^2^ respectively during 2010–2020. However, the SUHI intensity of Nanjing decreased from 4.60°C to 2.76°C over that period. In recent years, Nanjing has fallen out of the list of “big four stoves” for its improvement of thermal environment [59]. The upgrading of industrial structure and the improvement of atmospheric environment are the critical contributors for the improvement of thermal environment in Nanjing. From 2010 to 2020, Secondary industry as percentage to GDP in Nanjing dropped from 45.37% to 35.19%, while the portion of tertiary industry increased from 51.85% to 62.81%. The atmospheric environment of Nanjing has been significantly improved in this context. Its industrial sulfur dioxide emissions decreased from 115,507 tons in 2010 to 9709 tons in 2020, with a reduction rate of 91.59%; meanwhile, its carbon dioxide emissions and industrial dust emissions also decreased by 39.51% and 37.23%, respectively.

**Fig 6:**
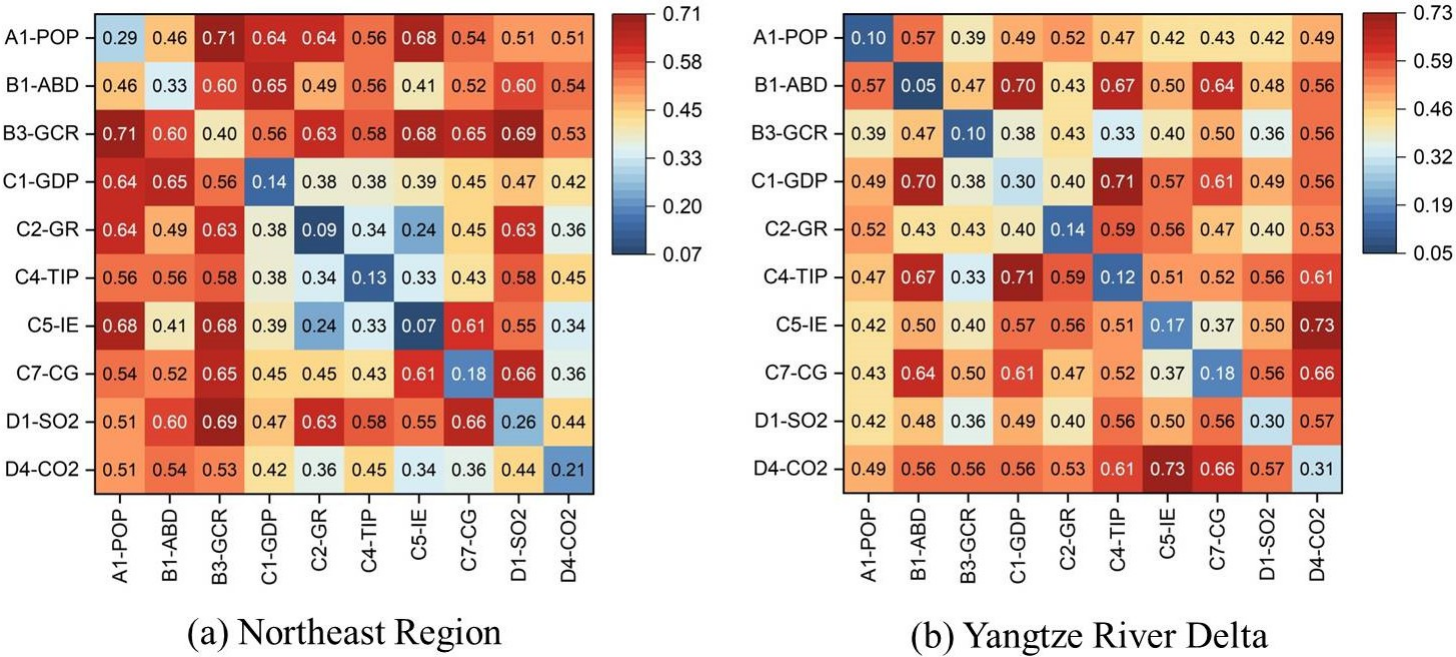
Results of the interaction detector. Notes: (a) Values in brackets are *q* statistics; (b) The full name of each indicator can be found in Table 1.

As is shown in Fig 6, there is a significant interaction effect between various socio-economic driving factors on the SUHI variations in NR and YRD, and most interaction types are dual-factor enhancement (Enhance, bi-) and nonlinear enhancement (Enhance, nonlinear-). These results indicate that the explanatory powers of drivers have been significantly improved (bigger *q* statistics) through interaction. In the NR, the interaction of urban population and green coverage rate has the highest explanatory power, and its *q* statistics rises to 0.7104. While in the YRD, the interaction of industrial enterprise quantity and carbon dioxide emission has the highest explanatory power, and its *q* statistics rises to 0.7265. In a word, the comprehensive effect of various socio-economic factors leads to the variation of SUHI intensity.

## 4. Discussion

This paper finds an interesting phenomenon: the SUHI effect is increasing in population shrinking region, while decreasing in growing region. Population agglomeration caused by rural-urban migration is one of the most important characteristic of urbanization [60, 61]. Dense population usually induces a series of eco-environmental problems in urban regions, including SUHI effect [62]. Therefore, it is often assumed that population agglomeration and urban expansion tend to intensify SUHI effect [63, 64]. Then, population shrinkage helps the mitigation of SUHI effect becomes a logical corollary. However, the results of this paper do not support this corollary. Our results indicate that the relationship between population change and SUHI effect is not significant, and population shrinkage will not naturally leads to the mitigation of SUHI effect. It’s true that population shrinkage provides rare opportunities for the ecological transformation in urban areas [65, 66], but more efforts are needed to turn these opportunities into reality. We agreed with the viewpoint that shrinking cities are not mirror images of growing cities [67]. Urban shrinkage is a complex and unique process, which does not bring about immediate and natural improvement of the urban ecological environment.

The results of driving mechanism analysis indicate that there are significant difference in the primary driving factors of SUHI variations in NR and YRD. Underlying surface change is the primary driver of the SUHI variation in the NR with the characteristic of population shrinkage. Growth-oriented has been the mainstream mode of urban development for quite a long time [68, 69]. In this context, how to push shrinking cities back to growth is the priority task for urban policymakers. These quick cures for shrinking cities, such as strengthening resource exploitation, accelerating infrastructure construction and increasing industrial investment, are natural choices for government decision-makers [70]. Therefore, urban shrinkage may leads to the urban spatial expansion and increasing resource consumption in the short term, thus cut down the expenditures in urban eco-environment construction, results in the further deterioration of urban environment [71, 72]. However, atmospheric environment change is the primary driver of the SUHI variation in the YRD with the characteristic of population growing. The YRD is one of the most developed and urbanized regions in China. Shanghai is the core city of the YRD region. In 2021, the urbanization rate of Shanghai reached 89.31%, ranking first in China; its per capita GDP is 175,420 yuan/person and ranking second in China. According to Northam’s theory of three-stage urbanization, the core feature of urbanization in the YRD is no longer the population migration from rural area to urban area, but the transformation of the leading industry from secondary industry to tertiary industry [73, 74]. The transformation and upgrading of industrial structure and economic growth not only reduce environmental pollution, but also provide more financial funds for the improvement of urban habitat environment, thus promoting the mitigation of SUHI effect. This logic is consistent with the theory of environmental Kuznets curve (EKC) [75, 76].

The significances and marginal contributions of this study are as follows: (1) it provides some new empirical evidences to the pending issues as regards the ecological transformation process of shrinking cities. Our results indicated that there is no definite conclusion between urban population change and SUHI intensity. For shrinking cities, population decline helps to cut down the individual consumption in transportation, resources and energy, thereby reducing urban anthropogenic heat emissions and contributing to the mitigation of SUHI effect [33, 34]. However, previous studies also found that the influences of economic size and development intensity on SUHI effect are greater than population change [40, 77]. If decision-makers still choose the extensive development path with the characteristics of dramatic spatial expansion and massive resource consumption, it will not only offset the benefits of SUHI mitigation brought by population shrinkage, but also leads to the continuous deterioration of the urban thermal environment. (2) It emphasizes the explanatory power of the historical urbanization process on SUHI effect. A large number of literatures discussed the impacts of some static driving factors on SUHI effect, such as underlying surface, climatic conditions, urban form and air pollution [29, 37, 78]. Regrettably, the impacts of socio-economic dynamic process on SUHI effect were rarely mentioned [54]. Although the SUHI effect is a physical geographical phenomenon, but in essence, it is a response of local thermal environment to several social-economic processes in the context of urbanization development [79], such as the evolution processes of urban population, industrial structure and resident consumption structure. Therefore, incorporating the SUHI effect into the urbanization process is an essential step to explore its deeper driving mechanism. This paper has made some preliminary attempts for this topic. But obviously, more efforts are needed to unravel the complex relationship between urbanization processes and SUHI effect. (3) It highlights the importance of regional scale analysis. It is widely accepted that urban shrinkage and SUHI effect are closely related to the individual characteristics of a specific city, such as natural conditions, demographics and industrial structure [2, 80]. Therefore, most previous studies were focused on city scale [81, 82]. However, growing literatures found that urban shrinkage and SUHI effect were spatially correlated [83, 84], which means the influences of regional macro background are significant too. Considering that the analysis results of urban shrinkage and SUHI effect at the city scale were greatly affected by the individual characteristics of each city, regional scale analysis is a hopeful option to surpass the limitations of traditional city scale analysis and reveal more general rules about urban shrinkage and SUHI effect.

There are some limitations in this research. (1) The difference of average surface temperatures between urban and rural areas is used to characterize SUHI intensity in this research. Although this processing mode is consistent with the traditional concept of SUHI effect [29], but the regional average value is too simple to reflect the range, amplitude and spatial pattern of urban thermal environment comprehensively and accurately. In addition, recent literature discovered that the amplitude and direction of SUHI intensity trends were significantly influenced by non-urban reference selections [85]. Therefore, the calculation of SUHI intensity still needs improvement in the future. (2) Annual LST data was utilized to analyze SUHI effect in this research. However, SUHI effect has significant diurnal and seasonal variations [86]. Ignoring these variations may introduce some uncertainty to the conclusions of this research. (3) This paper only discussed the interactions between two variables on SUHI effect, but failed to reveal the interactions among multiple driving factors.

## 5. Conclusion

This research compares the population change and SUHI effect between population shrinking region (NR) and growing region (YRD) in China, and explores their differences in driving mechanisms. The results indicated that there are significant differences in population changes and SUHI intensity between these two regions. About 72.22% of the cities in the NR were shrinking, while their SUHI intensities increased by an average of 1.69°C. On the contrary, the urban population in the YRD shows a linear growth trend and only 3 cities are shrinking, but their SUHI intensities decreased by 0.11°C on average. The results of bivariate Moran’s I index also indicated that the spatial correlation between the urban population changes and the variations of SUHI intensity are not significant in both population shrinking and growing regions. Furthermore, there are significant differences in the primary drivers of SUHI variations between these two regions. In the NR, underlying surface changes, including the changes of green coverage and built-up area, are the most important driving factors. However, atmospheric environment changes, such as carbon dioxide emission and sulfur dioxide emission, are the key drivers in the YRD. Northam’s theory of three-stage urbanization and environmental Kuznets curve hypothesis are powerful to explain these differences.

This research can be deepened from the following aspects: (1) considering the complicated relationship between population change and SUHI effect, more models are recommended to explore their nonlinear multiple response relationships in the future, such as system dynamic model, intermediate effect model and structural equation model. (2) More case regions around the world should be selected to further verify the conclusions of this paper in view of the diversity of global urban shrinkage and SUHI effect.

## Supporting information

S1 File (XLSX)
